# Natural products reverse cancer multidrug resistance

**DOI:** 10.3389/fphar.2024.1348076

**Published:** 2024-03-08

**Authors:** Jia-Yu Zou, Qi-Lei Chen, Xiao-Ci Luo, Davaadagva Damdinjav, Usama Ramadan Abdelmohsen, Hong-Yan Li, Tungalag Battulga, Hu-Biao Chen, Yu-Qing Wang, Jian-Ye Zhang

**Affiliations:** ^1^ Guangzhou Municipal and Guangdong Provincial Key Laboratory of Molecular Target & Clinical Pharmacology, The NMPA and State Key Laboratory of Respiratory Disease, School of Pharmaceutical Sciences and the Fifth Affiliated Hospital, Guangzhou Medical University, Guangzhou, China; ^2^ School of Chinese Medicine, Hong Kong Baptist University, Kowloon, Hong Kong SAR, China; ^3^ School of Pharmacy, Mongolian National University of Medical Sciences, Ulaanbaatar, Mongolia; ^4^ Deraya Center for Scientific Research, Deraya University, New Minia, Egypt; ^5^ Department of Pharmacognosy, Faculty of Pharmacy, Minia University, Minia, Egypt; ^6^ Ministry of Education Engineering Research Center of Tibetan Medicine Detection Technology, Xizang Minzu University, Xianyang, China; ^7^ The Affiliated TCM Hospital, Guangzhou Medical University, Guangzhou, China; ^8^ The Affiliated Qingyuan Hospital, Guangzhou Medical University, Qingyuan, China

**Keywords:** mechanism, natural product-derived compounds, cancer, multidrug resistance, MDR

## Abstract

Cancer stands as a prominent global cause of death. One of the key reasons why clinical tumor chemotherapy fails is multidrug resistance (MDR). In recent decades, accumulated studies have shown how Natural Product-Derived Compounds can reverse tumor MDR. Discovering novel potential modulators to reduce tumor MDR by Natural Product-Derived Compounds has become a popular research area across the globe. Numerous studies mainly focus on natural products including flavonoids, alkaloids, terpenoids, polyphenols and coumarins for their MDR modulatory activity. Natural products reverse MDR by regulating signaling pathways or the relevant expressed protein or gene. Here we perform a deep review of the previous achievements, recent advances in the development of natural products as a treatment for MDR. This review aims to provide some insights for the study of multidrug resistance of natural products.

## 1 Introduction

According to the World Health Organization (WHO) and International Agency for Cancer Research, there were approximately 19.3 million new cases of cancer in the past (Rudnicka et al., 2020), along with around 10 million cancer-related deaths (Sung et al., 2021). Cancer was one of the leading causes of mortality worldwide, and there were likely 28.4 million new cases by the year 2040 (Siegel et al., 2023). Chemotherapy could be confidently recommended for first-line treatment. MDR was a phenomenon in which cancer cells acquired cross-resistance to structurally and functionally related different types of anticancer drugs. However, the emergence of drug resistance made chemotherapy much less effective (Lu et al., 2022). Scientists have shown an interest in natural products (Zhang et al., 2020) because of their superiority in terms of rich resources, lack of negative side effects, and variety of components. Various biological actions are demonstrated by natural compounds, such as anti-tumor (Mans et al., 2000), anti-microbial (Chakrawarti et al., 2016; Martinelli et al., 2021; Papadochristopoulos et al., 2021), antioxidant (Arulselvan et al., 2016; Huang et al., 2017), anti-inflammatory (Arulselvan et al., 2016; Huang et al., 2017), anti-diabetic (Chen W. et al., 2019; Luo et al., 2019), anti-hypertensive (Fang et al., 2021; Gupta et al., 2022), anti-atherogenic (Penson and Banach, 2021), gastro-protective (Tamaddonfard et al., 2019), anti-platelets (Iqbal et al., 2022), anti-thrombotic (Csikós et al., 2021; Junren et al., 2021; Yen et al., 2022) vital effects on reversing MDR (Guo et al., 2017; Wang et al., 2018; Zhao et al., 2020).

The application of natural products in MDR has received much attention recently. This review examines the scientific advances on using natural products to treat tumors with MDR. We discuss the mechanisms of action of various kinds of natural products, including as flavonoids (Vissenaekens et al., 2022), alkaloids (Liu C. et al., 2019), terpenoids (Kuang et al., 2021), polyphenols (Luca et al., 2020), and coumarins (Xiaokaiti and Li, 2020; Giovannuzzi et al., 2022).

## 2 The mechanism of MDR

MDR refers to a cancer cell’s sensitivity to various anti-cancer medication therapies (Assaraf et al., 2019). Nevertheless, setting out the factors related to MDR could benefit the administration of antitumor drugs and curation (Lu et al., 2022). This paper describes the mechanisms of MDR, such as increasing drug efflux, altering drug targets, increasing DNA damage repair, MDR-related factors or signaling pathways, non-coding RNA (ncRNA)-mediated multidrug resistance, and autophagy and tumor microenvironment effects.

### 2.1 Increasing of drug efflux

Cancer cells exhibiting MDR typically increase the efflux of drug molecules, which reduces the chemosensitivity to anticancer medicines (Mohammad et al., 2018; Abdelfatah et al., 2021). Some genes associated with multidrug resistance in cancer are very unstable. For example, the CDK6 and CDK6-PI3K axes act synergistically in regulating ABCB1/P-gp-mediated expression of MDR (Zhang L. et al., 2022). It has been noted that long-term use of chemotherapy medicines increases the expression of the ABC transporter, thereby raising the risk of MDR. The ABC transporter by ATP hydrolysis move substrates outward against a concentration gradient (Jacobo-Albavera et al., 2021). For this reason, ATP-binding cassette sub-family B member 1, also known as P-glycoprotein (ABCB1/P-gp), ATP-binding cassette sub-family C member 1/Multidrug resistance-associated proteins (ABCC1/MRPs), and ATP-binding cassette sub-family G member 2/Breast cancer resistance protein (ABCG2/BCRP) are the most important drug transporters.

**FIGURE 1:**
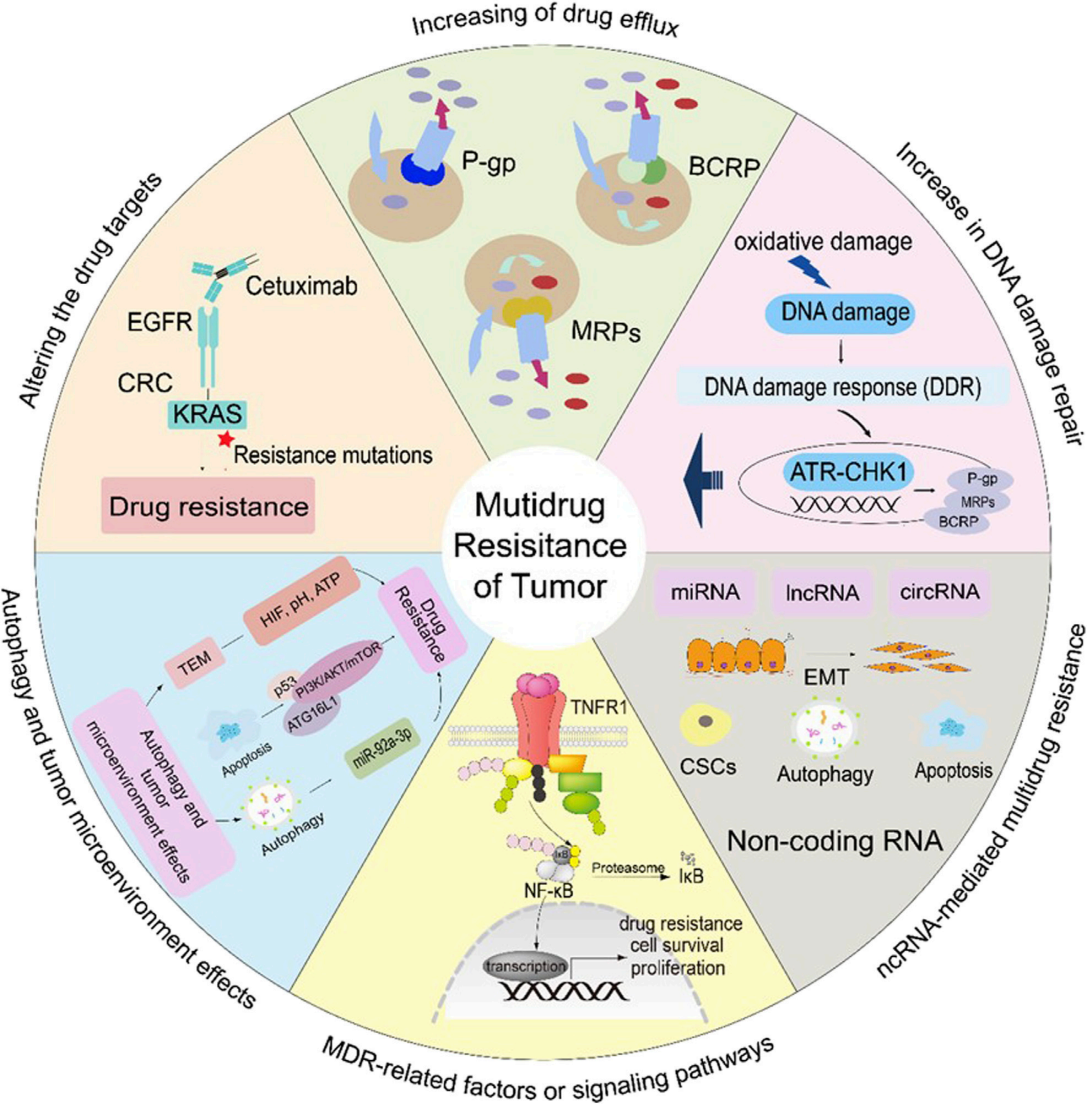
Mechanisms involved in cancer drug resistance.

P-gp is present in both healthy and malignant cells (Sarkadi et al., 2006). The transmembrane structural domain and the nucleotide-binding domain are the two primary structural domains that make up the P-gp transporter. The ABC protein’s structure may change upon binding to ATP, leading to concurrent change in the substrate binding site, which causes the substrate drug to be expelled from the cell. Different chemotherapeutic medicines bind to P-gp, which modifies the structure of P-gp and releases the drug into the extracellular space as ATP is digested (Assaraf et al., 2019) (Figure 1).

In various cancer types, such as lung, breast and prostate cancer, the expression of MRPs and BCRPs leads to resistance drugs (Ambudkar et al., 2003; Goel and Aggarwal, 2010). MRPs have the same structure and function as P-gp, but MRPs only transport medicines that have undergone glutathione (GSH) modification, and they have an impact on when pharmaceuticals are metabolically activated (Liu, 2009). Drugs are effluxed after MRPs create a complex with glutathione, glucuronate, or sulfate. Etoposide, doxorubicin (Dox), vincristine (VCR), and epirubicin (EPI) are among the drugs that are susceptible to MRPs (Liu, 2009). BCRP structure is homodimeric formed two-half transporters joined by a disulfide bond (Figure 1). Such drugs as mitoxantrone, topotecan, Dox, irinotecan, EPI, and flavopiridol are sensitive to BCRP (Ambudkar et al., 2003; Robey et al., 2007).

### 2.2 Altering the drug targets

Creizotinib resistance in lung adenocarcinoma, caused by an acquired mutation in the glycine-to-arginine substitution at codon 2032 in the structural domain of reactive oxygen species (ROS) proto-oncogene 1 (ROS1) kinase (Awad et al., 2013). Similarly, secondary EGFR mutations in the outer structural domain S492R lead to cetuximab resistance by preventing EGFR antibodies from binding to their target sites in colon cancer (Montagut et al., 2012) (Figure 1).

### 2.3 Increase in DNA damage repair

A major cause of tumor progression, is persistent genetic mutations have been occurring in the genome of cancer cells. The DNA damage response (DDR) is essential to protect cells from the large amount of oxidative damage to which they are periodically subjected by cellular damage (Groelly et al., 2023). DDR and cell cycle checkpoints are interlock signaling networks that can impede the cell cycle and transmission of genetic information to daughter cells to ensure genomic integrity (Maleki Dana et al., 2022). Cell cycle checkpoints enable the orderly progression of cell cycle events and avoid the development of genomic instability-related diseases such as cancer.

The G1 checkpoint is dysregulated in the majority of cancer cells, making them reliant on the S and G2 checkpoints, specifically ATR-CHK1. Cancer enhance their DNA repair system and promote survival by activating the ATR-CHK1 pathway. Chemotherapeutic drugs that target replicating DNA cells activate the ATR-CHK1 pathway (da Costa et al., 2023). Additionally, inhibitors of ATR-CHK1 have been reported to decrease the levels of P-gp (Ahmed et al., 2022).

Natural products, including curcumin, mangostin, resveratrol, and carnosine, block the G1-S phase but not affect the cell division cycle of proliferating healthy cells. Studies have shown that resveratrol, mangostin, and carnosine are agonists of ATR-CHK1, and kaempferol, curcumin, raffinose, and caffeine are inhibitors of ATR-CHK1, and these phytochemicals may help overcome tumor resistance (Ahmed et al., 2022). PU-1 is a sesquiterpenoid derived from Asteraceae that inhibits the growth of drug-resistant tumor cells through DNA damage, G2/M cell cycle blockade and apoptosis (Hegazy et al., 2021) (Figure 1).

### 2.4 MDR-related factors or signaling pathways

Some transcription factors aid in the growth and resistance to chemotherapy of drug-resistant cancer cells. The transcription factor Nrf2 aids to the resistance to chemotherapy and to proliferation of cancer cells by control the expression of cytoprotective and antioxidant enzymes (Ghareghomi et al., 2022; Panieri et al., 2022). Another important Five transcription factors that make up NF-κB can attach to responsive gene promoter regions and control biological activities such DNA transcription, cytokine generation, and cell survival. Apoptosis suppression, tumor cell proliferation, and treatment resistance are all caused by activated NF-κB. Numerous cancer forms exhibit abnormal factor regulation, which is exacerbated by the presence of the majority of anticancer medications in the cancer cells (Vlahopoulos, 2017; Xia et al., 2018) (Figure 1).

### 2.5 Non-coding RNA (ncRNA)-mediated multidrug resistance

Almost all cell processes, including transcription, proliferation, apoptosis, and differentiation, have been shown to be significantly regulated by non-coding RNAs (ncRNAs), particularly microRNA (Zou et al., 2017; Yang et al., 2021), long non-coding RNA (lncRNA) (Singh et al., 2022), and circular RNA. They also play a significant role in the regulation of cancer drug resistance (Leng et al., 2022). The expression of specific target genes, including Bcl-2, MDR1/ABCB1, and MRP1/ABCC1, which regulate apoptosis, autophagy, drug efflux, epithelial to mesenchymal transition (EMT), and cancer stem cells (CSCs), leads to the development of MDR. The interaction of ncRNAs with DNA, RNA, and proteins is the foundation for all of these mechanisms (Zhou et al., 2022a; Li et al., 2022) (Figure 1).

### 2.6 Autophagy and tumor microenvironment effects

#### 2.6.1 Autophagy

Autophagy breaks down damaged organelles and stops cells from accumulating too many anticancer drugs, which encourages the growth of multidrug resistance in cancer cells (Chang and Zou, 2020). However, autophagy in the cellular surroundings can also eliminate MDR tumor cells, thereby enhancing the efficacy of chemotherapy. This process is regulated by various autophagy-related pathways and components, including ATG16L1, ATG5, the PI3K/AKT/mTOR pathway, AMP-activated protein kinase (AMPK), the miR199a-5p/p62 axis, p53, TFEB, and NSAIDs (Debnath et al., 2023). The activation of autophagy facilitates the removal of drug-resistant tumor cells, thus improving the overall response to chemotherapy (Li et al., 2017; Rakesh et al., 2022; Xing et al., 2022) (Figure 1).

#### 2.6.2 Tumor microenvironment (TME)

Low extracellular pH, increased ROS concentrations, hypoxia, and the overexpression of certain proteases and factors are TME characteristics (Barkley et al., 2022). TME has its own blood supply, lymphatic and neurological systems, stroma, immune cells, and Extracellular Matrix (ECM) for each person with a specific tumor (Tiwari et al., 2022) (Figure 1).

Under hypoxic conditions, TME demonstrates chemoresistance and reduces drug-induced cytotoxicity, which encourages cancer growth and spread. For optimum activity, several anticancer medications like Cisplatin (DDP), etoposide, and gemcitabine require oxygen (Zhang W. et al., 2022). A key factor in hypoxia-induced chemoresistance is the HIF protein. Under hypoxic conditions, Hif-1α encodes P-gp and increases the expression of MRP1, BCRP, and LRP. Additionally, Hif-1α supports DNA repair processes and inhibits the effects of chemotherapeutic drugs (Emran et al., 2022).

Another important component of MDR is the control of pH. According to research, MCF-7 cells are more resistant to the effects of chemotherapeutic medicines when the extracellular pH is lower (Tavares-Valente et al., 2013). The ATP-rich tumor microenvironment is associated with cancer drug resistance, and cancer cells are able to take up extracellular ATP (eATP) through macrocytosis to increase intracellular ATP (iATP) levels and enhance drug resistance. Elevated iATP upregulated ABC transporter efflux activity in A549 and SK-Hep-1 cells, as well as PDGFRα and protein phosphorylation in the PDGFR-mediated Akt-mTOR and Raf-MEK signaling pathways in A549 cells (Wang et al., 2017).

## 3 Natural product—derived compounds overcome cancer drug resistance mechanisms

Based on various studies, natural compounds do work as multifunctional agents that can control the main causes of MDR. Below, we outline how certain natural compounds, such as flavonoids, alkaloids, terpenoids, polyphenols, and coumarins, can contribute to reducing MDR.

### 3.1 Flavonoids

**FIGURE 2:**
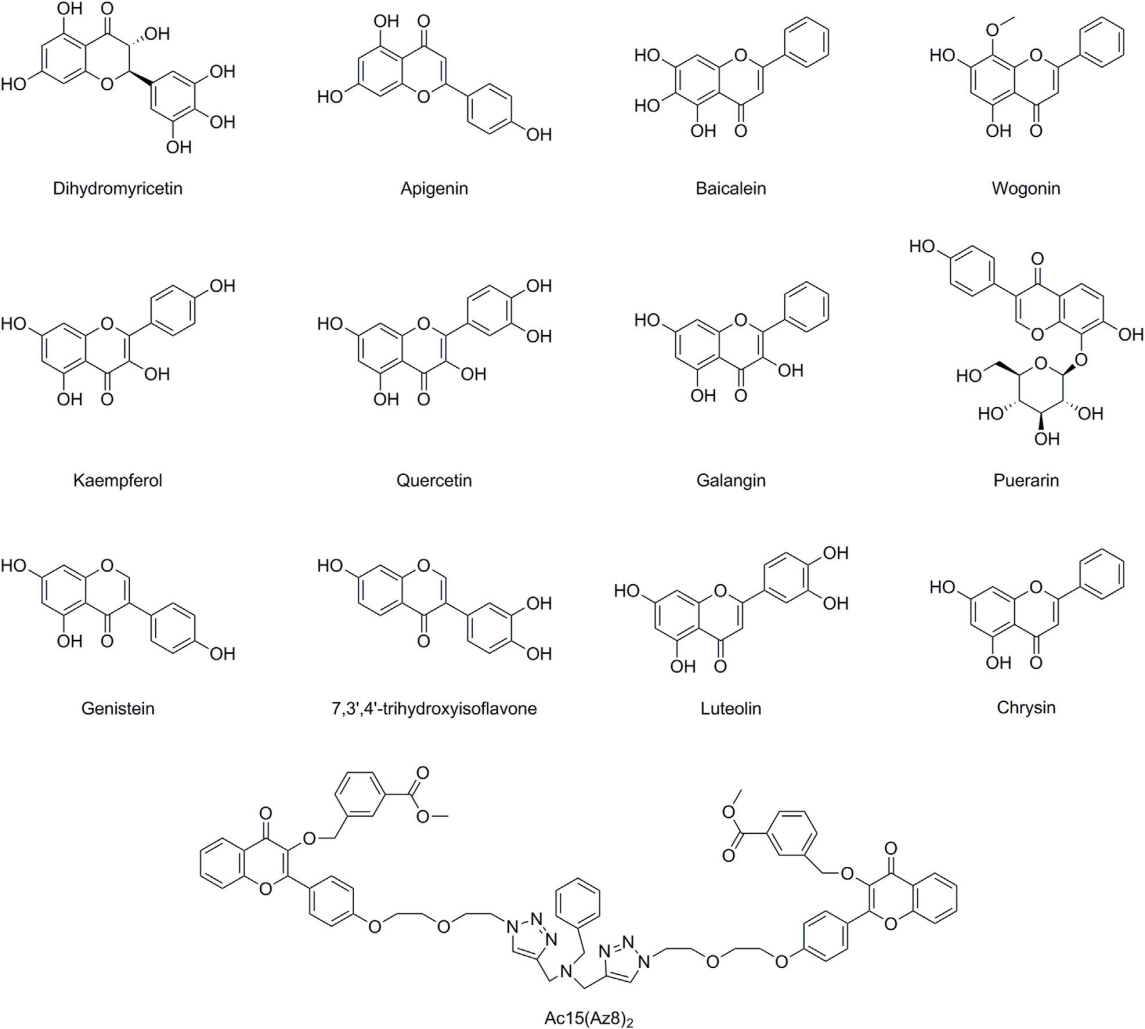
Chemical structures of Flavonoids having MDR modulatory activity.

In many different sections of plants, the most prevalent and significant secondary metabolites are flavonoids. Flavonoids, iso-flavonoids, and neo-flavonoids are the three types of flavonoids that can be distinguished by the presence of 2-phenylchromone ketone (Eichhorn and Efferth, 2012). These natural metabolites are frequently employed in clinical settings due to their anti-mutagenic, anti-oxidative, anti-inflammatory, and anti-carcinogenic effects as well as their ability to control important cellular enzymes (Kumar and Jaitak, 2019) (Figure 2; Table 1).

**TABLE 1:** The Effect of Natural Product-Derived Compounds on Cancer cells’ MDR.

	Compunds	Cells	Drug-resistant	Mechanism	ReF
Flavonoids	Dihydromyricetin	HCT116	OXA	Inhibiting MRP2 expression and promoter	Wang et al. (2021)
		HCT8	VCR	Inhibiting MRP2 expression and promoter	Wang et al. (2021)
		SGC7901	5-FU	Regulation expression of MDR1 mRNA and protein	Wang et al. (2021)
		OvCa	PTX; Dox	Activation of p53	Xu et al. (2017)
	Apigenin	Human CD44^+^ prostate CSCs	DDP	Downregulation of anti-apoptotic Bcl-2, sharping and surviving; upregulation of pro-apoptotic caspase-8, Apaf-1 and p53 mRNA	(Erdogan et al., 2016; Erdogan et al., 2017)
		A549; H1299	OXA/BLM	Elevate p53 and upregulate certain pro-apoptotic proteins	Chen et al. (2016)
	Acacetin	A549; H1299	OXA/BLM	Inhibiting of BCRP	(Punia et al., 2017; Singh et al., 2020)
	Baicalein	MDCKII	silymarin	Reverse P-gp	(Ferreira et al., 2018a; Ferreira et al., 2018b)
		BEL7402	Dox	Decreases P-gp and anti-apoptotic Bcl-xl expression	Li et al. (2018b)
		Thyroid carcinoma 8505c	TXT/PTX	Inhibiting of P-gp	(Meng et al., 2016; Park et al., 2018)
		MCF-7	PTX	Inhibiting of P-gp	(Meng et al., 2016; Park et al., 2018)
		Gastric AGS	5-FU	Inhibiting HIF-1α expression and Akt phosphorylation	Chen et al. (2015)
		A549	DDP	Inhibiting of PI3K/Akt/NF-κB signaling pathway	Yu et al. (2017b)
		PC3	TRvAIL	Induces ROS production and impact TME	Ye et al. (2019)
		breast cancer	TAM	Inhibiting HIF-1α-targeted glycolytic genes	Chen et al. (2021b)
	Wogonin	K562	A02/Dox	Inhibiting functional activity and expression of P-gp at both protein and mRNA levels	Xu et al. (2014)
		HCT116/	DDP	Inhibiting PI3K/Akt signaling pathway	He et al. (2013)
		Human breast cancer	sorafenib/Dox	Downregulation IGF-1R/AKT signaling pathway	Rong et al. (2017)
		A549	TRAIL	Induces ROS accumulation	Yang et al. (2013)
		HNC	DDP	Inhibited Nrf2 and glutathione S-transferase P	Ye et al. (2019)
	Kaempferol	OVCAR-3	DDP	Inhibits the mRNA levels of MRPs and cMyc	Amjad et al. (2022)
		HCT8-R	5-FU	Promotes the expression of miR-326, inhibit the process of glycolysis	Wu et al. (2022)
		CML	TRAIL	Enhance pro-apoptotic effects of anti-TRAIL antibody	Saraei et al. (2022)
		LS174-R	5-FU	Inhibiting reactive ROS; Modulated the expression of JAK/STAT3, MAPK, PI3K/AKT and NF-κB	Riahi-Chebbi et al. (2019)
	Quercetin	BEL	5-FU	Inhibits the functions and downregulates the expressions of P-gp, MRPs	Chen et al. (2018)
		Breast CSCs	Dox; PTX; VCR	Downregulates P-gp expression	Li et al. (2018c)
		MCF-7; 4T1; HCT116	DDP; etoposide	Regulates HIF-1α	Kim et al. (2012)
		Osteosarcoma 143B	DDP	Regulation miR-217-KRAS axis	Zhang et al. (2015b)
	Galangin	A2780/CP70; OVCAR-3	DDP	Increased the p53-dependent intrinsic and extrinsic apoptotic pathway	Huang et al. (2020)
		A549	DDP	Inactivating p-STAT3/p65 and Bcl-2 pathways	Yu et al. (2018)
	Puerarin	K562	Dox	Inhibited phosphorylation of Akt and JNK; Inhibition of NF-κB pathway; Downregulating MDR1	Liu et al. (2021b)
	Genistein	A549	radiotherapy	Downregulates the level of methylation in the Keap1 promoter region; Induces ROS production and impact TME	(Cort et al., 2016; Liu et al., 2016)
	7,3′,4′-trihydroxyisoflavone	HeLa	EPI	Down-regulating ABC transporters P-gp, MRPs	Hummelova et al. (2015)
	Luteolin	KKU-100	DDP	Inhibiting of Nrf2	Ye et al. (2019)
		HCT116-OX; SW620-OX	OXA	Inhibiting of Nrf2	Ye et al. (2019)
		A549	Dox; BLM	Inhibiting of Nrf2	Ye et al. (2019)
		MDA-MB 231	Dox	Inhibiting of Nrf2	Ye et al. (2019)
	Chrysin	MDA-MB-231	mitoxantrone	Inhibiting of P-gp	Ahmed-Belkacem et al. (2005)
		MCF-7	nitrofurantoin	Regulate BCRP; Stimulating ATPase	Ahmed-Belkacem et al. (2005)
	Ac15 (Az8)2	S1M180	topotecan	Inhibiting BCRP-ATPase activity and drug efflux	Chong et al. (2022)
Alkaloids	Mono- and di-carbamate	Human colon adenocarcinoma	Dox	Inhibition P-gp	Sancha et al. (2023)
	securinine	HepG2	Dox	Inhibition P-gp	Hou et al. (2023)
	Tetrandrine	Osteosarcoma	Dox	Changing the expression of MDR1 gene	Zhou et al. (2022b)
		Hep-2	VCR	Inhibit MDR1	Li et al. (2020a)
		YES-2	DDP	Inhibiting MRPs expression	Wang et al. (2018)
	Fangchinoline	Caco-2; CEM/DOX5000	Dox	Inhibition P-gp	Chan et al. (2021)
	Oxymatrine	CRC	5-FU	Induction of apoptosis; Suppressed expression of MRP1; Inactivated NF-κB signalling by decreasing phosphorylated p65	Chen et al. (2021a)
		CRC	OXA	Inhibiting of the NF-κB/PI3K/AKT/mTOR signal pathways	Chen et al. (2021a)
	Matrine	HT-29	OXA	Suppressed the expression of LRP and P-gp	Li et al. (2020b)
		K562	Dox	Promoting autophagy; Arresting the cell cycle	Li et al. (2020b)
	Rutaecarpine	MCF-7	Dox	Inhibiting the expression of P-gp	Zou et al. (2021)
		A549	PTX	Inhibiting the expression of P-gp	Zou et al. (2021)
	Voacamine	U-2 OS-DX	Dox	Interfered with the P-gp-mediated drugs export acting	Condello et al. (2020)
Polyphenols	Resveratrol	AML -2/DX300	Dox	Inhibiting expression of MRP1	Li et al. (2018c)
		HCT 116	Dox	Inhibition P-gp	Wang et al. (2020)
		K562	Dox	Inhibition of the PI3k/Akt/mTOR pathway	Khan et al. (2020)
		MCF-7	Dox	Regulation miR-122-5p; Regulation of Bcl-2 and CDKs	ALkharashi (2023)
		Human oral cancer CAR	DDP	Induces expression of mRNA autophagy-related genes, including Beclin-1, Atg5, Atg12, and LC3-II; Enhance phosphorylation of AMPK	Chang et al. (2017)
	Epigallocatechin-3-gallate	KB-A1	Dox	Inhibition P-gp	Li et al. (2018a)
		OVCAR3; SKOV3	DDP	Increasing the expression of CTR1; Inhibiting the degradation of CTR1	Wang et al. (2015)
		Ovary cancer; NSCLC	DDP	Increasing ROS generation and CTR1 expression; Regulation of ERK1/2/NEAT1 pathway	Chen et al. (2020a)
	Curcumin	A2780cp	DDP	Demethylate in the promoter region of MEG3; Downregulation of miR-214	Zhou et al. (2017)
		HL-60	Dox	Regulation the HOTAIR/miR-20a-5p/WT1 pathway	Liu et al. (2021a)
	EF24	Ovarian	DDP	Overexpression of p53 and p21 proteins; Induced apoptosis; Activating PTEN phosphorylation inhibiting Akt	Ibáñez Gaspar and McMorrow (2023)
	GO-Y030	K562	mitoxantrone	Inhibit BCRP	Murakami et al. (2017)
Terpenoid	β-Elemene	A549	ER	Inhibition P-gp	Lin et al. (2018)
		MCF-7	Dox; Doc	Regulation miRNA29a, miRNA222; Inhibiting the PI3K–AKT signaling pathway	Hu et al. (2019)
		A549	DDP	Decreasing mitochondrial membrane potential and increasing intracellular ROS concentrations	Liskova et al. (2021)
		SPC-A1	DDP	Promoting Beclin-1	Li et al. (2021)
Coumarins	PFC	HCT-116	irinotecan	Inhibits BCRP-mediated drug-transport function	Kokubo et al. (2021)

Dihydromyricetin (DMY), a naturally occurring flavonoid derived from Vitis heyneana, a traditional Chinese medicine plant. By reducing MDR1 mRNA and protein expression to 5-FU cytotoxicity, DMY decreases MRP2 expression and its promoter activity in HCT116/Oxaliplatin (OXA)and HCT8/VCR cells as well as sensitized SGC7901/5-FU cells (Wang et al., 2021). In order to reestablish chemosensitivity in CRC cells, DMY suppresses the Nrf2/MRP2 signaling pathway (Wang et al., 2021). DMY also activates p53 and induces apoptosis in paclitaxel (PTX)- and Dox-resistant OvCa cells (Xu et al., 2017).

Apigenin (API), a common dietary flavonoid, inhibits P-gp and BCRP, increasing cellular uptake of anticancer drugs such as Dox or TXT in MDR (Noorian et al., 2022). API is reported to suppress cell growth, clonogenicity, and invasiveness in CSCs. In human CD44^+^ prostate CSCs, API can upregulate caspase-8, apaf-1, and p53 mRNA expression, downregulate Bcl-2, sharpin, and survival, and increase the effectiveness of DDP (Erdogan et al., 2016; Erdogan et al., 2017). API suppresses STAT3, Akt, and MAPK in glioblastoma multiforme U87MG and U373MG cells in other CSCs(Kim B. et al., 2016). A549/OXA/bleomycin (BLM) and H1299/OXA/BLM cells may undergo a large rise in apoptosis as a result of API’s ability to activate p53 and pro-apoptotic proteins (Chen et al., 2016). Acacetin, an O-methylated API, inhibits the MDR1 gene at the mRNA level in NSCLC model cell lines A549/OXA/BLM and H1299/OXA/BLM (Punia et al., 2017; Singh et al., 2020).

Wogonin (WOG), a compound known as 5,7-dihydroxy-8-methoxyflavone, is found in fruits, vegetables, and certain medicinal plants. It possesses a wide range of biological activities, including anti-cancer, anti-inflammatory, and the treatment of bacterial and viral infections (Huynh et al., 2020). By preventing P-gp’s expression and functional activity, WOG sensitizes Dox-resistant K562/A02 cells (Xu et al., 2014). By lowering the expression of HIF-1α in HCT116/DDP cells, WOG suppresses the PI3K/Akt signaling pathway and increases cytotoxicity when combined with medicines like DDP, Dox, and PTX (He et al., 2013). WOG potentiates apoptosis and inhibits autophagy by regulating AKR1C1/1C2 and TNF-α (Huynh et al., 2017b). WOG blocks the IGF-1R/AKT signaling pathway in human breast cancer, increasing the cytotoxicity of sorafenib and Dox (Rong et al., 2017). In human osteosarcoma CSCs with anti-CD133 (Yang et al., 2022), WOG behavior demonstrate apoptosis through downregulating MMP-9 expression, which inhibits mobility and stops cell renewal (Huynh et al., 2017a). In response to internal and external stressors brought on by ROS, Nrf2 acts as a transcription factor by up-regulating antioxidant proteins (Leinonen et al., 2014; Sajadimajd and Khazaei, 2018). WOG induces ROS accumulation and further sensitizes TRAIL-induced apoptosis in A549 cells (Yang et al., 2013). Additionally, WOG increases ROS buildup, which increases intracellular ROS, and inhibits Nrf2 nuclear translocation via inactivating NF-κB (Ye et al., 2019). In DDP-resistant HNC cells, WOG inhibited Nrf2 and glutathione S-transferase P, increasing intracellular ROS (Kim E. H. et al., 2016).

Kaempferol (KAE), 3,4′,5,7-tetrahydroxyflavone, is a secondary metabolite found in many plants, and traditional medicines. KAE can inhibit ABCB1/P-gp through enhancing the capacity of ATPase (Felice et al., 2022), and also as an ABCG2/BCRP substrate, it can inhibit ABCG2/BCRP upregulation. KAE appears to have potential synergies with DDP, inhibits the mRNA levels of MRPs and cMyc in OVCAR-3 cells (Amjad et al., 2022). KAE promotes the development of miR-326, suppresses the process of glycolysis, and the resistence of HCT8-R cells to 5-Fluorouracil (5-FU) (Wu et al., 2022). KAE may increase the capacity of chronic myeloid leukemia (CML) cells to withstand the pro-apoptotic effects of anti-TRAIL antibodies (Saraei et al., 2022). Additionally, in human LS174-R colon cancer cells that are resistant to 5-FU, KAE suppresses reactive oxygen species and modifies the expression of JAK/STAT3, MAPK, PI3K/AKT, and NF-κB (Riahi-Chebbi et al., 2019).

Quercetin can be found in numerous fruits such as apples, berries (such as blueberries and cranberries), citrus fruits (such as oranges and lemons), and grapes. It is also present in vegetables such as onions, broccoli, kale, and tomatoes. Additionally, quercetin can be found in leaves of plants such as tea leaves and grains like buckwheat. In 5-FU-resistant BEL/5-FU cells, QUE would prevent ABCB1/P-gp and MRPs from functioning and expressing (Chen et al., 2018). Dox, PTX, VCR, and QUE combined treatment substantially reduces ABCB1/P-gp expression and eliminates breast CSCs (Li S. et al., 2018). Additionally, quercetin suppresses colorectal and breast CSCs (Azizi et al., 2022). QUE can control HIF-1α, which re-sensitizes 4T1 cells, MCF-7/Dox cells, and HCT116 cancer cells to DDP and etoposide (Kim et al., 2012). Through the miR-217-KRAS axis, QUE boosts osteosarcoma 143B cells’ sensitivity to the chemotherapy drug DDP (Zhang X. et al., 2015).

Collateral sensitivity (CS) involves the exploration of medications that specifically induce a higher level of cytotoxicity in MDR cells compared to the original non-resistant cells (Efferth et al., 2020). Galangin (GA), which has been extracted from the root of *Alpinia galanga*, has greater inhibitory effects on MDR cells (Lorendeau et al., 2014), and demonstrates collateral sensitivity (Lorendeau et al., 2017). A study found that GA increased the p53-dependent apoptotic pathway in ovarian cancer cells A2780/CP70/DDP and OVCAR-3/DDP, favorably inducing apoptosis compared to normal ovarian cells (Huang et al., 2020). GA through inactivating p-STAT3/p65 and Bcl-2 pathways attenuates DDP-induced resistance in A549 cells (Yu et al., 2018).

An isoflavone called puerarin (PU) was isolated from the plant *Pueraria lobata (Willd.) Ohwi*. There is a study by Li et al. which shows that PU suppresses Akt and JNK phosphorylation and promotes death in K562/Dox cells, as well as that autophagy makes tumor cells more resistant to anticancer drugs (Liu Q. et al., 2021). By inhibiting the NF-κB pathway and reducing MDR1 expression, PU sensitized K562/Dox cells (Liu Q. et al., 2021).

The primary daidzein metabolites genistein (IFG) and 7,3′,4′-trihydroxyisoflavone (TDI), which are present in fruits, nuts, and soy, also exhibit anti-Nrf2 and anti-ROS properties. The degree of methylation in the Keep1 promoter region is controlled by IFG, which decreases nucleus transcription and raises ROS in A549 cells (Cort et al., 2016; Liu et al., 2016). IFG and radiation are both effective in increasing cell apoptosis in A549 cells. TDI downregulation of ABCB1/P-gp and MRPs leads to a significant increase in EPI accumulation and attenuation of EPI resistance in HeLa cells (Hummelova et al., 2015). The methylation of some cancer-related genes may be hijacked by cancer cells to promote tumorigenesis. Research shows that IFG (0.5–10 μM) may lower the level of methyl DNA transference (MNDF). IFG (0.5–10 μM) significantly decreased the methylation of the Estrogen receptor β (ER-β) promoter in prostate cancer (PCa), ER-β which has an inducing effect on PCa cellular metabolism (Ji et al., 2022). IFG regulates caspase-3 and p38MAPK pathways and induces apoptosis in PC3 prostate cancer cells (Song et al., 2020).

Luteolin (LU), 3′,4′,5,7-Tetrahydroxyflavone, is a compound that is abundantly found in leaves and aromatic flowering plants. It possesses anti-inflammation, anti-allergy, and anti-cancer properties, and can function as either an antioxidant or a pro-oxidant biochemically (Lo et al., 2012). Additionally, LU has the power to dramatically decrease Nrf2 and enhance the cytotoxicity of DDP in KKU-100 cholangiocarcinoma cells; HCT116/OXA and SW620/OXA cells; A549/BLM/Dox cells and in MDA-MB 231/Dox cells. By increasing the expression of the epithelial biomarker E-cadherin, LU can reverse EMT. It also suppresses Hif-1α signaling in cervical cancer cell lines to prevent the invasivity from being activated (Imran et al., 2019). In pancreatic cancer cells, LU induces apoptosis by blocking the K-RAS/GSK-3β/NF-κB signaling pathway (Imran et al., 2019).

Honey, propolis, and the passion flower Passiflora caerulea all contain chrysin, which is a 5,7-dihydroxyflavone. Chrysin has the power to stop the efflux of ABCB1/P-gp from MDA-MB-231/mitoxantrone cells and regulate the transport of nitrofurantoin through ABCG2/BCRP from BCRP-overexpressing MCF-7 cells. Moreover, chromatin sensitizes BCRP-transfected cells via stimulating ATPase (Ahmed-Belkacem et al., 2005).

Due to the pseudo-dimeric shape of ABC transporters, Synthetic compounds were synthesized from natural flavonoids using a “click chemistry” method to efficiently produce a variety of triazole-bridged homo- and heteroflavonoid dimers. Ac15(Az8)2, a flavonoid dimer, inhibits BCRP potently, safely, and specifically. Through the inhibition of BCRP-ATPase activity and drug efflux in S1M180/topotecan cells, Ac15(Az8)2 restored intracellular drug accumulation, according to mechanistic investigations (Chong et al., 2022).

### 3.2 Alkaloids

**FIGURE 3:**
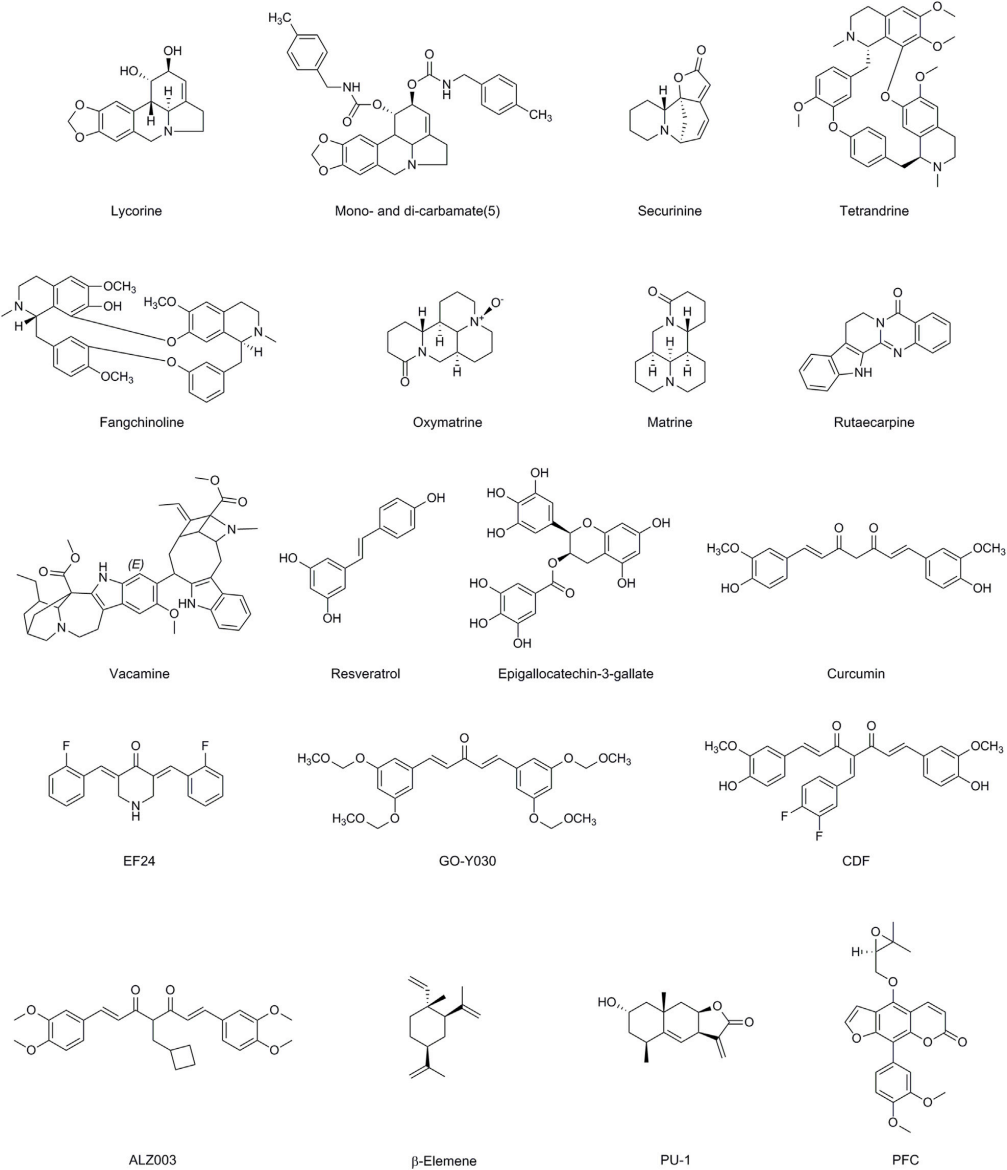
Chemical structures of Alkaloids, Polyphenols, Terpenoids and Coumarins having MDR modulatory activity.

Alkaloids are also secondary metabolites that are found in a variety of plants, fungi, and bacteria worldwide. The presence of one or more basic nitrogens, often in a heterocyclic ring, and significant pharmacological activity characterize an alkaloid. Their P-gp inhibitory function is likewise influenced by the basic nitrogen atoms (Gonçalves et al., 2020) (Figure 3; Table 1).

Lycorine (LYC) is a commonly used alkaloids extracted from the bulb of Lycoris radiate, recognized for its various biological effects, which include anticancer, antiviral, antibacterial, and anti-inflammatory activities (Roy et al., 2018). TCRP1 is a new candidate for a human gene that is associated with chemotherapeutic resistance. It is expressed extensively in various types of cancer cells and is associated with chemotherapeutic resistance (Liu X. et al., 2019). LYC lowers the levels of tongue cancer and Hepatocellular Carcinoma (HCC) cells TCRP1 protein by promoting the degradation pathway of TCRP1 protein, which translates into repression of Akt/mTOR signaling, and therefore activates apoptotic and autophagic abilities (Yu H. et al., 2017). Bioinformatics analysis revealed that upregulation of FABP5 expression facilitates acute myeloid leukemia (AML) cell viability, protects AML cells from apoptosis. LYC downregulated the expression levels of FABP5 and its target PPARγ, impaired AML cell viability, and triggered apoptosis (Liang et al., 2023). By performing extracellular, cytoplasmic, and nuclear roles, HMGB1 is crucial for stress signaling as well as for the activation of autophagy. Because autophagy is inhibited in human bone marrow CD138 primary myeloma cells and multiple myeloma (MM) cell lines, LYC-induced proteasomal degradation of HMGB1 inhibits the activation of the MEK-ERK signaling pathway and Bcl-2 phosphorylation declines, leading to the constitutive association of Bcl-2 with Beclin-1 (Roy et al., 2016). LYC inhibits EGF-induced JAK/STAT signaling as well as various downstream STAT3 targets, such as cyclin D1, Bcl-2, Bcl-xL, matrix metalloproteinase 2 (MMP2), and the EMT promoter Twist, which lowers prostate cancer cell line proliferation, migration, invasion (Hu et al., 2015). Amaryllidaceae alkaloids were investigated as MDR reversibles in human colon cancer cells by derivatizing alkaloid hydroxyl groups into mono- and di-carbamates. Di-carbamates that contain phenethyl or benzyl moieties were found to be more potent inhibitors than verapamil. The collateral sensitivity of a number of derivatives also suggested a dual role in reversing P-gp-mediated MDR (Sancha et al., 2023).

Securinega alkaloids (SA) are indolizidine alkaloids derived from the Asian plant *Securinega suffruticosa*’s leaf and root. SA Several activities have been documented, including antiproliferative activity, leukemia differentiation induction activity, MDR reversal activity, antimalarial activity, and antibacterial activity (Hou et al., 2023). Recent studies have found altering securinine at the C15 sites increases the ability to reverse drug resistance caused by several drugs, whereas derivatives with a bivalent mimic attached to the C15 site increase the ability to induce differentiation and reverse drug resistance caused by P-gp. Mechanism investigations indicated MDR reversal action in HepG2/Doxvia reduction of P-gp function against Dox (Hou et al., 2023).

The bisbenzyl isoquinoline alkaloid tetrandrine (TET), which was isolated from the Chinese plant *Stephania tetrandra* (Han-Fang-Chi), has anticancer effects because it inhibits cell proliferation and induces apoptosis (Zhou et al., 2022b). Previous research has shown that TET and its derivatives can reverse MDR caused by osteosarcoma/Dox. TET may significantly increase intracellular chemotherapeutic drug concentration by changing the expression of the MDR1 gene and P-gp, and can be used in combination with chemotherapy drugs to significantly inhibit P-gp expression (Zhou et al., 2022b). TET inhibits drug efflux caused by MDR1 overexpression and has anti-MDR action in Hep-2/VCR cells (Li Y. et al., 2020). TET decreased transporter protein mRNA and protein levels suppress MRP overexpression in MDR human esophageal squamous carcinoma YES-2/DDP cells and epidermis-like k2–mrp1 cancer cells (Wang et al., 2018). Molecular dynamics simulations were employed to design OY-101, a novel chemical compound derived from the modification of natural TET. OY-101 demonstrated selective and potent inhibition of P-gp (Zeng et al., 2023). Fangchinoline (FAN) is a prominent bisbenzylisoquinoline (BBIQ) alkaloid derived from the roots of *S. tetrandra* in the Menispermaceae family that consists of two benzylisoquinoline units joined by oxygen bridges. FAN has been shown to reverse MDR in Caco-2 and CEM/Dox5000 cancer cells when combined with Dox (Chan et al., 2021).

Oxymatrine (OMA) and matrine (MA), as natural compounds derived from *Sophora flavescens*, have been reported to possess a wide range of pharmacological properties, including anti-inflammatory, antiviral, anti-tumor, and immunomodulatory effects. In addition to considerably reversing cellular MDR, enhancing apoptotic induction, suppressing MRP1 expression, and inactivating NF-κB signaling by lowering phosphorylated p65, OMA alone or in conjunction with 5-FU significantly reversed cellular MDR (Du and Shim, 2016). One of the signaling pathways responsible for 5-FU resistance in CRC is NF κB. Combining OMA and OXA improved both the *in vitro* and *in vivo* anticancer effects of OXA in CRC cancer cells. These additive effects were attained by suppressing the NF κB and PI3K/AKT/mTOR signaling pathways, which decreased the treatment resistance of CRC cancer cells. OMA is known to inhibit the NF κB pathway, so its combination with OXA could provide an enhanced therapeutic benefit (Chen M. H. et al., 2021). LRP, or lung resistance protein, is a cytoplasmic vault protein that plays a role in both the vesicular sequestration of drugs in the cytoplasm and their translocation from the nucleus to the cytoplasm. MA reversed drug resistance in OXA-resistant HT-29/OXA cells, increased HT-29/OXA cells’ sensitivity to OXA in a dose-dependent manner, and significantly decreased LRP and P-gp expression in HT-29/OXA cells at the mRNA and protein levels. In K562/Dox cells, MA causes a dose-dependent stoppage of the cell cycle at the G0/G1 phase, which promotes autophagy (Li Z. et al., 2020).

Rutaecarpine (Rut), a bioactive alkaloid found in *Evodia rutaecarpa*, has been associated with various pharmacological effects such as analgesic, anticancer, and anti-inflammatory properties. On P-gp-overexpressing MCF-7/Dox and A549/PTX cells, Rut dose-dependently improved the effectiveness of Dox, PTX, and colchicine. Since the ubiquitination pathway plays a major role in protein degradation, the E3 ubiquitin ligase MARCH8 is an ABCB1/P-gp substrate. MARCH8 interacts with ABCB1/P-gp to promote ubiquitination and degradation (Zhang et al., 2023). Rut can boost MARCH8 expression, which encourages the degradation of ABCB1/P-gp (Zou et al., 2021). Castration-resistant prostate cancer (CRPC) is largely brought on by androgen receptor splice variation 7 (AR-V7). Rut restores the susceptibility of castration-resistant prostate cancer to anti-androgen therapy *in vitro* and *in vivo* by specifically inducing AR-V7 protein degradation via K48-linked ubiquitination (Liao et al., 2020).

A bisindole alkaloid known as voacamine (VOA) was discovered in the *Voacanga* and *Peschiera* species of the Apocynaceae family. VOA possesses a variety of biological qualities, including antibacterial action, resistance to Plasmodium falciparum, strong neuroprotective activity against Alzheimer’s disease, and the ability to inhibit the mutagenicity brought on by several genotoxic substances (Theissinger et al., 2023). VOA was an effective substrate for P-gp and acted as a competitive antagonist to obstruct P-gp-mediated drug export. When VOA was administered to U-2 OS-DX/Dox cells, laser scanning confocal microscopy (LSCM) studies showed a disorganizing effect on microtubules (Condello et al., 2020).

### 3.3 Polyphenols

A massive family of 10,000 plant compounds known as polyphenols is mostly present in fruits, green and black tea, coffee, red wine, chocolate, and seeds (Zhang Y. et al., 2022). Polyphenols frequently have three-membered flavan ring structures. In the body, free radicals primarily fall into two categories: ROS and reactive nitrogen species (RNS). The involvement of the B rings in scavenging ROS/RNS and the capacity of hydroxyl groups connected to benzene rings to donate a hydrogen atom or an electron to free radicals are essential elements of the mechanism underpinning polyphenol action. Cell death, EMT, ROS, DNA repair procedures, CSCs, and epigenetics [such as MicroRNAs (miRNAs)] are some other targets that polyphenols affect in order to combat chemoresistance in cancer cells (Wang et al., 2022) (Figure 3; Table 1).

Numerous cancers, including bladder, prostate, breast, lung, glioblastoma, colon, and ovarian, are affected by resveratrol (RES) in terms of apoptosis (Ren et al., 2021). Under the regulation of MRP1, various endogenous and xenobiotic substrates are absorbed and eliminated. Due to the elevated levels of MRP1 gene expression in Dox-resistant acute myeloid leukemia (AML)-2/DX300 cells, its expression may lead to a decrease in drug cellular absorption (Chen J. et al., 2019). RES with Dox decreases Dox IC50 from 0.96 ± 0.02 M to 0.52 ± 0.05 M in HCT 116 colorectal cancer cells, increases Dox intracellularly, and inhibits the efflux action of P-gp (Wang et al., 2020). Inhibitory the PI3K/Akt/mTOR pathway causes the inhibitory impact of RES over P-gp in K562/Dox cells (Khan et al., 2020). RES recognize miR-122-5p and regulates Bcl-2 and CDKs, resulting in the chemosensitization of Dox-resistant breast cancer MCF-7 cells (ALkharashi, 2023). In DDP-resistant human oral cancer CAR cells, RES therapy promotes the expression of autophagy-related genes, such as Beclin-1 Atg12 and LC3-II, at the mRNA level and increases AMPK phosphorylation. This results in regulated autophagy and pro-apoptosis-related signals to be send (Chang et al., 2017). RES dramatically reduced the activation of tumor-promoting factors (NF-κB, MMP-9, CXCR4) and epithelial-to-mesenchymal transition-factors (increased vimentin and slug, decreased E-cadherin) in TNF-induced activation of CRC cells by preventing EMT and CSC formation (Buhrmann et al., 2018). RES treatment results in elevated levels of DNA topoisomerase-II (TOPO), an enzyme that is commonly found in malignancies and plays a crucial role in maintaining DNA structure during transcription and DNA replication. Furthermore, the RES-treated group exhibited significantly higher levels of Topo-II compared to other groups. It appears that RES promotes drug-induced DNA damage in Dox-resistant PUMC-91/Dox cells (Skok et al., 2020; Choi et al., 2022). 5-FU resistant (5-FU-R) cells may exhibit resistance to the DNA-damaging chemical 1,3-bis(2-chloroethyl)-1-nitrosourea (BCNU). However, when RES and BCNU are combined, it is enhance the sensitivity and induce DNA damage in 5-FU-R cells (Choi et al., 2022).

A significant polyphenolic component of green tea called epigallocatechin-3-gallate (EGCG) has several beneficial properties, including the capacity to lower stress, regulate metabolism, prevent cancer, and offer protection from various diseases. In drug-resistant KB-A1 cells, EGCG has been found to alter P-gp activity and increase intracellular Dox concentration (Tang et al., 2017; Li H. et al., 2018). The primary copper influx transporter CTR1 is in charge of copper’s resistance to platinum. Because EGCG raises CTR1 expression at the mRNA and protein levels in ovarian cancer cells and upregulates the rapid DDP-induced degradation of CTR1, OVCAR3 and SKOV3 ovarian cancer cells are more vulnerable to DDP when it is present (Wang et al., 2015). Ovarian cancer and non-small-cell lung cancer (NSCLC) cells were made more susceptible to DDP when supplemented with EGCG by increasing ROS generation and CTR1 expression through activating the ERK1/2/NEAT1 pathway (Chen A. et al., 2020). Through suppression of the Bcr/Abl oncoprotein and control of its downstream p38-MAPK/JNK and JAK2/STAT3/AKT pathways, EGCG was able to decrease cell proliferation and cause apoptosis in CML (Xiao et al., 2019). The DDP-induced DNA damage is substantially repaired by the 5′-3′ structure-specific endonuclease ERCC1/XPF. A DNA-endonuclease incision test based on fluorescence was used to identify (Heyza et al., 2018). EGCG exhibits inhibitory effects on colorectal CSCs and lung CSCs. It downregulates the activation of the Wnt/β-catenin pathway (Chen et al., 2017; Fujiki et al., 2018).

Curcumin (Cur) is an active polyphenolic pigment obtained from the rhizomes of *Curcuma longa*. Cur is typically used for its antioxidant, anti-inflammatory, wound-healing, and anti-carcinogenesis qualities that halt the onset or progression of cancer (Tomeh et al., 2019). The inhibition of ABC family transporters by Cur in various cancer cells causes drug accumulation within cancer cells (Dong et al., 2023). Cur is a helpful medicine when used in conjunction with significant chemotherapeutic medications to combat MDR. Cur causes DNA damage in several cell lines and inhibits particular DNA repair enzymes. Rad51-dependent homologous recombination is a crucial DNA repair pathway that enables cancer cells to develop resistance to medications that target tumor DNA damage. However, Cur has been found to lower the expression of Rad51, leading to DNA damage in cancer cells (Zhao et al., 2018; Wong, 2021). According to reports, Cur is a powerful DNA hypomethylation agent that inhibits DNMT1 activity by covalently attaching to and inhibiting the catalytic thiol group of cysteine (C1226) (Wu et al., 2016; Tong et al., 2020). By preventing the expression of DNMT1, Cur slows down cell growth and triggers apoptosis in hepatocellular carcinoma (Liu et al., 2017). Numerous varieties of multidrug resistant cancer cells were revealed to have improper NF-κB regulation. The expression of genes regulated by NF-κB is decreased as a result of Cur’s suppression of NF-κB activity and prevention of NF-κB binding to DNA (Zhou et al., 2017). Cur reduces IκBα kinase activity in human head and neck squamous cell carcinoma cell lines, which blocks NF-κB activity. By blocking the PI3K/AKT pathway, which reduces NF-κB expression, Cur increases the sensitivity of cancer cells to treatment (Mortezaee et al., 2019a; Villegas et al., 2021; Abadi et al., 2022). Cur may demethylate in the MEG3 promoter area in the A2780cp ovarian cancer cell line, which downregulates miR-214 and, indirectly, lowers DDP resistance (Zhou et al., 2017). Liu et al. proposed a potential regulatory network involving HOX transcript antisense RNA (HOTAIR), miR-20a-5p, and Wilms’ tumor 1 (WT1). Their findings demonstrated that Cur inhibits the levels of WT1 in human acute myeloid leukemia cells (HL-60) or HL-60/Dox cells. They also observed that the suppression of miR-20a-5p, resulting in increased WT1 expression, attenuated the effect of Cur on the resistance of leukemia cells to Dox. These results indicate that Cur inhibits the resistance of tumor cells to Dox by targeting the HOTAIR/miR-20a-5p/WT1 axis. Cur slows the proliferation of cancer cells, as evidenced by the downregulation of NF-κB in mantle cell lymphoma, oral MCF-7, and non-small-cell lung carcinoma (Meiyanto et al., 2014). Additionally, the activation of redox processes within cells leads to the generation of ROS, which upregulates the apoptotic receptors on the membrane of tumor cells (Mortezaee et al., 2019b). Cur decreases tumor cell growth and increases apoptosis by upregulating the expression and activity of p53, attenuating the regulation of antiapoptosis PI3K signaling and MAPKs to boost endogenous ROS generation, and overexpressing antiapoptosis genes such Bcl-2 (Mortezaee et al., 2019b). By controlling oxidative stress, modulating fibrosis, activating SIRT1, and encouraging cellular apoptosis, Cur may kill MCF7/TH, HCT116R, and A549/Dox cancer cells (Gabr et al., 2022). A therapeutic target, nicotinamide N-methyltransferase (NNMT) has a variety of effects on CRC aggressiveness and 5-FU resistance (Abadi et al., 2022). Cur has the ability to reduce NNMT and p-STAT3 expression. Particularly in CRC cell lines with significant NNMT expression, Cur may also lessen ROS generation, G2/M phase cell cycle arrest, and cell growth (Keyvani-Ghamsari et al., 2020; Gabr et al., 2022).

Cur plays a wide range of roles in cancer cells’ multidrug resistance, but its clinical application is challenging. Cur’s weak solubility, minimal absorption, restricted tissue distribution, and quick metabolism are its significant drawbacks. Generating novel Cur analogues is one method to solve these issues. EF24 is a developed Cur analog. By suppressing NF-κB, HIF-1α, controlling the creation of ROS, and controlling important genes by miRNA, EF24 prevented cancer cells from going through the cell cycle and caused apoptosis (Bisht et al., 2016; Ibáñez Gaspar and McMorrow, 2023). EF24 makes ovarian cancer resistant cells more susceptible to DDP by causing an overexpression of the p53 and p21 proteins in the G2/M checkpoint. In DDP-resistant cells, it also induces apoptosis by promoting PTEN phosphorylation and inhibiting Akt, a resistance cell (Su et al., 2023). Go-Y030, EF24 analog of Cur, can stop K562/mitoxantrone cells from producing mitoxantrone and pheophorbide A from ABCG2/BCRP (Murakami et al., 2017). Another Cur homologue with better bioavailability is CDF. Pancreatic cancer cells underwent apoptosis and had their NF-κB activity inhibited when CDF and gemcitabine were combined (Lee et al., 2018). Additionally, PTEN expression was increased while overexpressed miR-21 expression was decreased by CDF, which prevented cells resistant to gemcitabine from undergoing cellular arrest (Lee et al., 2018). The androgen receptor (AR) in prostate cancer has been reported to promote treatment resistance. *In vitro* and *in vivo*, a Cur analog named ALZ003 significantly lowers the survival of TMZ-sensitive and -resistant glioblastoma by triggering FBXL2-mediated AR ubiquitination, which leads to its degredation (Bott et al., 2016; Chen T. C. et al., 2020).

### 3.4 Terpenoids and Coumarins

#### 3.4.1 Terpenoids

Terpenoid is a class of natural compounds that is both extensively studied and structurally diverse. Terpenoids are classified into monoterpenoids (C10), sesquiterpenoids (C15), diterpenoids (C20), sesterterpenoids (C25), triterpenoids (C30), tetraterpenes (C40), and polyterpenes based on the number of isoprene units present in the parent structure. Terpenoids display numerous medicinal benefits, such as hypoglycemia, liver protection, antibacterial, anti-inflammatory, and anti-tumor characteristics (Kumar and Jaitak, 2019) (Figure 3; Table 1).

The sesquiterpene chemical β-Elemene (β-ELE), derived from *Curcuma Rhizoma*, exhibits properties such as inhibiting cell proliferation, arresting the cell cycle, inducing cell death, and reversing MDR in chemotherapy. β-ELE may be able to overcome drug resistance in human NSCLC A549/ER cells that are resistant to erlotinib (ER) *in vitro* by lowering P-gp expression, suppressing P-gp dependent drug efflux, and increasing intracellular concentrations of anticancer drugs (Lin et al., 2018). MiRNAs found in exosomes, function in the mechanism of intercellular communication and modify chemosensitivity. The intercellular transfer of certain miRNAs is partly responsible for the MDR of tumor cells. The ability of MDR Breast Cancer Anti-Estrogen (BCA) cells to propagate drug resistance is determined by the exosomes they produce. Recent research has demonstrated that β-ELE influences exosome content, affects the expression of certain MDR-related miRNAs, and reduces the exosome-mediated transmission of drug resistance, thereby enhancing the cells’ capacity to overcome MDR (Zhang J. et al., 2015). In the exosomes of multidrug-resistant gastric cancer cells, miR-1323 is significantly expressed, which encourages EMT of gastric cancer sensitive cells and enhances their capacity for invasion and migration (Tan et al., 2021). The expression of Cbl-b is likewise suppressed by miR-1323, resulting in the attenuation of drug resistance in cancer cells with MDR. By reversing the drug resistance and metastasis generated by exosomes, β-ELE reduces the ability of SGC7901 cells to resist drugs and migration (Tan et al., 2021). In terms of internal miRNA-29a and miRNA222, human MCF-7 cells that were resistant to Docetaxel (MCF-7/Doc) and Dox (MCF-7/Dox), respectively, were significantly downregulated by β-ELE. The tumor cells’ medication resistance was overcome by suppression of the PI3K-AKT signaling pathway. PTEN, a common tumor suppressor gene that blocks the PI3K-AKT signaling pathway, is the two miRNAs’ target gene and is significantly increased after miRNA-29a and miRNA222 are downregulated (Hu et al., 2019). A549/DDP cells underwent apoptosis when exposed to β-ELE because it decreased the mitochondrial membrane potential and increased intracellular ROS levels which may cause apoptosis and mitochondrial damage (Liskova et al., 2021) By increasing Caspase-3 protein expression, β-ELE may be used to overcome gastric cancer resistance (Low et al., 2021). β-ELE can additionally reduce chemoresistance in lung cancer by inhibiting the paracrine activities mediated by cyclin-dependent kinase inhibitor P21, which are regulated by CDK8. SPC-A1/DDP, a DDP-resistant lung cancer cell line, is more susceptible to apoptosis and medication treatment when β-ELE which is accomplished via fostering Beclin-1-induced autophagy (Li et al., 2021).

PU-1 is a sesquiterpene with the α-methylene-γ-lactone moiety that has been isolated from numerous plant species of the genera Inula and the genus Pulicaria. It might have anti-inflammatory and anti-cancer effects. In CCRF-CEM leukemic cells, the PI3K/AKT pathway prevents the development of drug-resistant tumor cells by causing DNA damage, obstructing the G2/M cell cycle, and triggering apoptosis. Resazurin reduction tests showed that PU-1 suppressed this pathway (Hegazy et al., 2021).

#### 3.4.2 Coumarins

Coumarins are a class of organic compounds that are widely distributed in nature, including in plants such as fruits, vegetables, and herbs. They are characterized by a benzene ring fused to an alpha-pyrone ring. The phenylfurocoumarin derivative (R)-9-(3,4-dimethoxyphenyl)-4-((3,3-dimethyloxiran-2-yl)methoxy)-7H-furo [3,2-g]chromen-7-one (PFC) in HCT-116/BCRP colon cancer cells, drastically lowers the IC50 of SN-38 while inhibiting ABCG2/BCRP-mediated drug transport function. Additionally, in the ABCG2/BCRP-overexpressing HCT-116/BCRP cell xenograft mice model, PD-stimulated ABCG2/BCRP-mediated ATP hydrolysis reduced irinotecan resistance without resulting in toxicity (Kokubo et al., 2021). In a recent study, fifteen sesquiterpene coumarins were extracted and purified from various Ferula species and tested for their ability to reverse MDR. The study found that the sesquiterpene coumarins enhanced Dox cytotoxicity in MCF-7/Dox cells, which are Dox-resistant derivatives of MCF-7 cells that overexpress the P-gp protein. Further analysis of the structure-activity relationship of these sesquiterpene coumarins indicated that ring-opened drimane-type compounds, specifically farnesiferol B, farnesiferol C, and lehmferin, exhibited the most potent inhibitory effects on P-gp pump efflux. These compounds could be considered as lead scaffolds for future modifications to improve their efficacy in reversing MDR (Kasaian et al., 2015) (Figure 3; Table 1).

## 4 Conclusion

Drug resistance is usually the reason why cancer treatments fail, despite major advancements in the manufacturing of new chemotherapeutic medications. Researchers have started taking conventional treatments like natural components more into consideration recently because of their reduced cost and adverse effects. Numerous studies have shown that the efficacy of natural products can influence various elements of cancer medication resistance. As described in this review, natural products have significant effects in overcoming drug resistance and enhancing the efficacy of chemotherapy. Natural products modulate inhibition of ABC transporters, increase in DNA damage, regulate ncRNA-mediated multidrug resistance, activate apoptotic cells, regulate the expression of metabolic enzymes, and chemosensitivity in various types of cancers both *in vitro* and *in vivo*.

Natural products are easily obtainable, but improving their qualities requires considering factors such as biological availability, biocompatibility, and half-life to enhance clinical efficacy and reduce risks to patients. To create more efficient antitumor drug delivery systems, the structure of tumor drugs is utilized to optimize drug properties. Additionally, computer-aided drug design can help in the development of natural products by predicting drug targets, modifying drug structure, and predicting toxicity. The focus of cancer therapeutic medication research is now on creating safe and effective natural products with fewer harmful side effects.

Patients who have developed medication resistance are being treated in clinical settings using a multi-targeted approach. For example, MET amplification causes cells to bypass EGFR and activate the PI3K/AKT pathway, allowing them to resist the effects of the EGFR inhibitors and develop resistance. For patients who have both MET amplification and EGFR mutations, the best treatment strategy is to use a dual-targeted EGFR/MET regimen to inhibit both EGFR and MET. To delay the development of resistance in KRAS-G12C patients, combination therapy is likely to become the preferred approach. When compared to mono-therapeutic regimens, combination techniques can significantly increase a therapy’s safety and effectiveness, particularly if the combined medications have different modes of action. The creation of biomarker-driven therapeutics is the result of the identification of certain resistance mechanisms. Both targeting tactics that anticipate the selection of resistant and combinatorial strategies that target multiple resistance nodes are being considered. Clinical research on natural products currently available indicate that they are primarily adjuvant medications. For instance gemcitabine coupled with celecoxib and curcumin in the treatment of patients with pancreatic cancer, in a prospective phase II trial, the safety and efficacy of curcumin (2000 mg/day in four capsules of 500 mg each) and gemcitabine (10 mg/m^2^) were evaluated in 44 patients with advanced and metastatic pancreatic cancer. The data showed a median progression-free survival and overall survival of 8.4 and 10.2 months, respectively. These findings suggest that the combination of gemcitabine and curcumin phytosome can safely and effectively increase the rate of response to first-line treatment for advanced pancreatic cancer. Natural substances may act as sensitizing agents in the therapy of cancer when used in combination, according to a growing body of research. In order to bring more effective treatments for reversing MDR to the clinic, it is crucial to uncover more potent and less dangerous chemicals and research their pharmacological mechanisms using cutting-edge technology, such as high-throughput screening methods, next-generation sequencing, advanced imaging techniques, and computational modeling. These technologies enable researchers to analyze complex biological systems at a molecular level, identify novel drug targets, and design innovative therapeutic strategies. By harnessing cutting-edge technology, scientists can accelerate the discovery and development of new drugs that can overcome multidrug resistance and improve patient outcomes. In order to encourage their clinical application and offer fresh ideas for the treatment of tumor drug resistance, this review covers the research progress of natural products in tumor drug resistance during the past few years.
